# Acceptability of financial incentives for maintenance of weight loss in mid-older adults: a mixed methods study

**DOI:** 10.1186/s12889-018-5136-z

**Published:** 2018-02-13

**Authors:** Bronwyn McGill, Blythe J. O’Hara, Anne C. Grunseit, Adrian Bauman, Dale Osborne, Luke Lawler, Philayrath Phongsavan

**Affiliations:** 1 Prevention Research Collaboration, Sydney School of Public Health, University of Sydney, Sydney, NSW 2006 Australia; 2 Charles Perkins Centre, John Hopkins Drive, University of Sydney, Sydney, NSW 2006 Australia; 3 The Australian Prevention Partnership Centre, Ultimo, NSW 2007 Australia; 4 Osborne Research Services, Rozelle, NSW 2039 Australia; 5 Prima Health Solutions, PO Box 7468, Warringah Mall, NSW 2100 Australia

**Keywords:** Financial incentives, Weight loss maintenance, Lifestyle modification, Formative research, Health insurance

## Abstract

**Background:**

Health insurers worldwide implement financial incentive schemes to encourage health-related behaviours, including to facilitate weight loss. The maintenance of weight loss is a public health challenge, and as non-communicable diseases become more prevalent with increasing age, mid-older adults could benefit from programs which motivate weight loss maintenance. However, little is understood about their perceptions of using financial incentives to maintain weight loss.

**Methods:**

We used mixed methods to explore the attitudes and views of participants who had completed an Australian weight loss and lifestyle modification program offered to overweight and obese health insurance members with weight-related chronic diseases, about the acceptability and usefulness of different types of financial incentives to support weight loss maintenance. An online survey was completed by 130 respondents (mean age = 64 years); and a further 28 participants (mean age = 65 years) attended six focus groups.

**Results:**

Both independent samples of participants supported a formalised maintenance program. Online survey respondents reported that non-cash (85.2%) and cash (77%) incentives would be potentially motivating; but only 40.5% reported that deposit contracts would motivate weight loss maintenance. Results of in-depth discussions found overall low support for any type of financial incentive, but particularly deposit contracts and lotteries. Some participants expressed that improved health was of more value than a monetary incentive and that they felt personally responsible for their own health, which was at odds with the idea of financial incentives. Others suggested ongoing program and peer support as potentially useful for weight loss maintenance.

**Conclusions:**

If financial incentives are considered for mid-older Australian adults in the health insurance setting, program planners will need to balance the discordance between participant beliefs about the individual responsibility for health and their desire for external supports to motivate and sustain weight loss maintenance.

**Electronic supplementary material:**

The online version of this article (10.1186/s12889-018-5136-z) contains supplementary material, which is available to authorized users.

## Background

Behavioural economics identifies patterns of behaviour which characterise the way individuals make decisions and aims to motivate behaviour change through interventions that positively influence decision making [1, 2]. The use of financial incentives incorporating insights from behavioural economics has become more common in promoting healthy lifestyles in recent years [3–5]. Health insurers worldwide have started to implement health promoting financial incentive schemes [6–8] and there is interest among Australian health insurers to follow suit [9]. As approximately half (57.1%) of all Australians have private health insurance cover [10], this is an important setting to encourage healthier lifestyle behaviours targeting chronic disease prevention and management.

Health promotion interventions that utilise financial incentives have been shown to positively impact lifestyle related behaviours such as smoking, healthy eating, alcohol consumption and physical activity [11, 12]. They have also been shown to have a positive effect on weight loss in adults, with the first six months of a weight loss program being most effective [13]. Research also suggests that weight loss is rarely sustained after the incentive is removed [14, 15] and that no significant changes in weight loss or weight loss maintenance have been reported at 12 and 18 months follow up [16]. Additionally, it is unclear whether financial incentives are effective at eliciting long-term behaviour change and health outcomes [17]. Despite the lack of long-term effects of financial incentives on weight loss maintenance in adults, interventions investigating the use of financial incentives for the maintenance of weight loss continue to add to the literature in this field [18, 19]. With increasing life expectancy [20] and prevalence of non-communicable disease among older Australian adults increasing [21], there is merit in investigating whether increasing motivation for behaviour change through financial incentives in this target population may be a cost-effective approach to lowering the burden of negative outcomes of lifestyle-related diseases. As most of the current research has been in the general adult population, the effectiveness of financial incentives among older adults in changing health behaviours is unclear [22].

A successful and effective financial incentive health intervention needs to be acceptable to the target population, healthcare professionals and policy makers [23–25]. Research investigating the acceptability and perceived usefulness of financial incentives has shown mixed results for different demographic groups in different contexts. For example, a study exploring the acceptability of financial incentives to motivate health-enhancing behaviours among US and UK adults reported that they may be less acceptable than similarly effective medical interventions for drug use, mental health problems, weight loss and smoking cessation [26]. Negative attitudes towards using financial incentives have been reported for health promoting financial incentives in UK adults, blood pressure management in hypertensive US adults and quitting smoking in pregnant Australian women [27–29]. Men and younger adults have been identified as more likely to prefer an incentive to no incentive, compared with women and older adults [25]. Effectiveness and cost-effectiveness of financial incentives have been consistently identified as key determinants for acceptability of financial incentives for healthy behaviours [30]. Additionally, financial incentives which are considered fair, which benefit individuals and wider society and which are delivered to suitable recipients are likely to be acceptable [30]. However, a lack of qualitative data limits a full understanding of the strength of feelings towards health promoting financial incentives [30]. Among older adults, a systematic review of financial incentives for healthy lifestyle and disease prevention suggests poor acceptability and a lack of trust about the use of financial incentives for health-related behaviour change [22].

There is a paucity of literature pertaining to the use of financial incentives in Australian lifestyle interventions involving mid-older adults despite their increased risk for the development of chronic disease [21]. Furthermore, the perceived effectiveness and acceptability of using financial incentives in weight loss maintenance among mid-older Australian adults has not been explored. As part of a project investigating the use of financial incentives for weight loss maintenance in mid-older private health insurance members, this formative research used a mixed methods approach to explore the attitudes and views of participants who had completed an intensive weight loss and lifestyle modification program regarding the acceptability and usefulness of different financial incentives to support the maintenance of their weight loss and a healthy lifestyle.

## Methods

### Design

We used a partially mixed methods sequential design where qualitative data was dominant over quantitative data [31]. Specifically, an initial quantitative data collection phase (Study A - surveys) was conducted independently of a subsequent qualitative phase (Study B - focus groups). The mixed method approach allowed multiple perspectives on the use of financial incentives for weight loss maintenance to be considered with integration of data occurring at the stage of data interpretation [31, 32]. Ethics approval was granted by the University of Sydney Human Research Ethics Committee (project numbers: 2016/772 and 2017/146).

### Participants and recruitment

The current study comprised participants who completed the Healthy Weight for Life (HWFL) program. HWFL is an Australian intensive weight loss and lifestyle modification program provided to private health insurance members who have a Body Mass Index ≥28 kg/m^2^ and a chronic disease (osteoarthritis, cardiovascular disease or type 2 diabetes) [33]. The program delivers three six-week phases over 18 weeks. Each phase includes a portion-controlled eating plan (including KicStart™ meal replacements), recommendations for gentle activity and personalised symptom and progress tracking, along with personal motivation, support and advice from the centralised HWFL Care Support Team (HWFL team) via phone, SMS, email, and mail. More than 80% of participants complete the program [34] and participants lose an average of 7% of their baseline weight over the 18 weeks [35]. The HWFL program is not an incentive-based program, but the service provider and one health insurer were interested in exploring the potential use of financial incentives as part of a long-term weight loss maintenance program. The current study was conducted in two parts. Two samples of participants were drawn from two populations of HWFL participants (see following section for details) and invited to take part in either Study A or Study B. Participants of study A did not participate in study B.

#### Study a: Quantitative phase

The study population comprised members of three major private health insurers with osteoarthritis who participated in and completed the HWFL program Australia-wide from August to December 2015 (*n* = 524). HWFL participants received an email invitation in July 2016 to complete an online survey exploring follow-up weight loss maintenance options. A reminder email was sent one week after the initial invitation. Participants completed the survey online after confirming consent to participate online.

#### Study B: Qualitative phase

This study comprised a subset of all HWFL participants - members of HCF, an Australian private health insurance company - who had completed the HWFL program in the year prior to November 2016 and lived in the Sydney metropolitan area (*n* = 175). These participants were invited by email in early November 2016 to take part in focus groups about the use of financial incentives to assist with weight loss maintenance. Interested participants provided consent to be contacted by a researcher who explained the study further, and scheduled a suitable time to attend a focus group. Written consent was obtained from each participant before the commencement of each focus group.

Focus group methodology was used to explore a range of participant perspectives about the use of financial incentive for weight loss maintenance [36]. An experienced and independent facilitator (author DO) conducted the focus groups, each averaging 80 min. Focus group interviews were audio-recorded and transcribed verbatim. Study investigators (BM and BOH) observed each group through a one-way mirror and made field notes. Following each interview, participants self-completed a pen-and-paper survey where they completed demographic information as well as weight status (whether they had maintained, gained or lost weight since completing the program).

### Data collection

Study A: Participants were asked about their interest in receiving ongoing support to maintain their weight loss, and in particular the use of financial incentives. The online survey (see Additional file 1) also collected socio-demographic data such as gender, age and postcode.

Study B: A semi-structured discussion guide (see Additional file 1) was used to explore HCF participants’ experiences of the HWFL program, experiences since completing the program, thoughts about a maintenance program and descriptions of and thoughts about different types of financial incentives (see Table 1).

**Table 1:** Description of types of financial incentives

Cash incentive	An agreed amount of cash, provided after a period of time or at a number of time points if weight loss is maintained
Non-cash incentive	An incentive with a monetary value but that is not cash, provided after a period of time or at a number of time points if weight loss is maintained (e.g. retail or gift voucher, gym membership, etc.)
Deposit contract	Make a monetary deposit which is refunded at certain time intervals or at the end of a period of time if you maintain your weight (i.e. a commitment to maintain your weight)
Matched deposit contract	Same as deposit contract, plus the deposit amount is matched if weight loss is maintained (i.e. “double your money”)
Lottery*	Go into a draw to win either a cash or non-cash ‘prize’ or incentive

*The lottery incentive was included for Study B but not for Study A

### Analysis

Socio-demographic variables and type of financial incentives were analysed descriptively and proportions are presented by study. To determine support for the use of financial incentives, the level of agreement on a five point-scale from strongly disagree to strongly agree with how much different types of financial incentives (Table 1) would motivate weight loss maintenance were collapsed into either Agree/Strongly Agree or Neutral/Disagree/Strongly Disagree. Postcodes were used to define Socio-Economic Indexes for Areas (SEIFA) as a measure of area socio-economic status [37]; and Accessibility-Remoteness Index of Australia Plus (ARIA) as an indication of geographical location remoteness [38]. Analyses were undertaken in IBM SPSS Statistics 21 [39].

For the qualitative analysis, study investigator BM listened to recordings to check and correct each transcript for accuracy, before importing the data to NVIVO 11 qualitative analysis software [40]. Study investigators BM and BOH collaboratively developed an initial coding frame and independently coded two common interviews. Common themes were identified using a thematic inductive approach, and were generated from the interview content rather than being predetermined [41]. Thematic content was then compared for consistency and coding discrepancies resolved by discussion. The coding frame was modified accordingly and the remaining transcripts coded by BM. Further analysis by BM identified recurrent themes through an iterative process exploring the reasoning for participant responses to major concepts discussed. These were then tested against the data and interpretations refined in consultation with BOH and AG. Themes were checked across participants’ reported gender and weight status. The analysis reached thematic saturation as no new additional information arose despite the different demographic composition of the groups.

## Results

### Participants

Study A: Of 524 participants invited to be involved in the online survey, 130 (24% response rate) completed the survey.

Study B: Of 175 HCF members invited, 58 (33% response rate) consented to be contacted about attending a focus group. Successful telephone contact was made with 57 members and a suitable focus group time arranged with 33 members. Twenty eight participants (16% response rate) attended six focus groups comprising one male-only (*n* = 6), two female-only (*n* = 4; *n* = 2) and three mixed gender groups (n = 4; n = 6 and n = 6).

#### Participant characteristics

More females (67.7%) than males completed Study A, and slightly more males (53.6%) completed Study B (Table 2). The mean age of participants for Study A was 64 years (SD = 7.8; range = 43–80 years old) and for Study B was 65 years (SD = 8.5; range = 47–79 years old). Consistent with recruitment, the majority of Study B participants were from the most advantaged socio-demographic areas, whereas Study A participants were more evenly distributed across the different socio-demographic areas. For example, 60.7% (*n* = 17) of Study B participants and 23.1% (*n* = 30) of study A participants were from areas of highest advantage. For Study B participants, 25% (*n* = 7) reported losing more weight since completing the program, 35.7% (*n* = 10) reported staying the same and 39.3% (*n* = 11) reporting putting on weight. This information was not available for Study A participants.

**Table 2:** Demographic characteristics of Study A and Study B participants

		Study A (*N* = 130)		Study B (*N* = 28)	
		n	%	n	%
Gender	Female	88	67.7	13	46.4
	Male	42	32.3	15	53.6
Age Group	40–44	2	1.5	0	0
	45–54	10	7.7	4	14.3
	55–64	53	40.8	6	21.4
	65–74	54	41.5	15	53.6
	75+	11	8.5	3	10.7
SEIFA^a^	1st Quintile (most disadvantaged)	18	13.8	2	7.1
	2nd Quintile	24	18.5	0	0
	3rd Quintile	23	17.7	3	10.7
	4th Quintile	35	26.9	6	21.4
	5th Quintile (most advantaged)	30	23.1	17	60.7
ARIA^b^	Major city	85	65.4	27	96.4
	Inner regional	20	15.4	1	3.6
	Outer regional/ Remote/Very Remote	25	19.2	0	0
Weight status^c^	Lost more weight	–	–	7	25.0
	Stayed the same weight	–	–	10	35.7
	Put on weight	–	–	11	39.3

^a^SEIFA provides a summary of people living in an area representing the general level of socio-economic disadvantage of all the people in that area

^b^ARIA is calculated and is based on the road distance from a locality to the closest service centre

^c^Weight status was self-reported (at the time of focus groups) and collected on from Study B participants only

Study A included the online survey and Study B was the focus group study

### Quantitative results (study a)

Study A respondents were mostly (93.9%, *n* = 122) supportive of the idea of a maintenance program following the HWFL program. When asked whether financial incentives three to six months after program completion would be helpful with weight loss maintenance, more than half (56.9%, *n* = 74) responded positively (Table 3). The majority of these respondents (85.2%, *n* = 63) perceived that non-cash incentives would motivate weight loss maintenance. Agreement for motivation by cash incentives was 77% (*n* = 57). Deposit contracts were perceived as being potentially motivating to 40.5% (*n* = 57) of respondents and matched deposit contracts for 48.7% (*n* = 36) (Table 4). Of Study A respondents who agreed that financial incentives would be helpful with weight loss maintenance (*n* = 74), the amount of money which would be sufficiently incentivising varied. Specifically, one third (33.8%, *n* = 25) answered that $50 would be enough; and 25.7% (*n* = 19) that $200 or more would be needed. The types of acceptable non-cash incentives also varied, but 32.4% (*n* = 24) preferred vouchers for food products, and a quarter preferred general gift vouchers (24.3%, *n* = 18) or reward points (23.0%, *n* = 17).

**Table 3:** Study A participant response to helpfulness of financial incentives

Financial incentives would be helpful	*N*	%
Yes – very helpful	48	36.9
Yes – somewhat helpful	26	20.0
No – not helpful	30	23.1
No opinion	26	20.0

**Table 4:** Study A participant responses to financial incentive questions

	*N*	%
Motivation: non-cash reward		
Strongly agree	48	64.9
Agree	15	20.3
Neither agree or disagree	9	12.2
Disagree	2	2.7
Strongly disagree	0	0.0
Motivation: cash reward		
Strongly agree	37	50.0
Agree	20	27.0
Neither agree or disagree	13	17.6
Disagree	3	4.1
Strongly disagree	1	1.4
Motivation: deposit contract		
Strongly agree	20	27.0
Agree	10	13.5
Neither agree or disagree	19	25.7
Disagree	14	18.9
Strongly disagree	11	14.9
Motivation: matched deposit contract		
Strongly agree	31	41.9
Agree	5	6.8
Neither agree or disagree	18	24.3
Disagree	12	16.2
Strongly disagree	8	10.8
Motivation: amount of money		
$50	25	33.8
$100	14	18.9
$150	7	9.5
$200	12	16.2
More than $200	7	9.5
Other	9	12.2
Deposit if goal not met		
Donated to a charity of participant’s choice	57	77.0
Donated to any charity	10	13.5
Other	7	9.5
Motivation: non-cash rewards		
Reward points	17	23.0
Gym membership	4	5.4
General gift vouchers	18	24.3
Vouchers for leisure-related products	6	8.1
Vouchers for food products	24	32.4
Other	5	7.0

Notes: *N* = 74, this analysis excludes participants who answered “no opinion” or “no” to the question “*Would the provision of a financial incentive (cash or non-cash) 3–6 months after the completion of HWFL be helpful to keep you on track with your weight management?”*

### Qualitative results (study B)

Two broad topics were discussed during the focus groups: participants’ general impressions of the option of a weight loss maintenance or ‘booster’ program and the nature of financial incentives for weight loss maintenance, which included exploring different types of financial incentives. Further, a number of recurrent themes were identified regarding what participants considered to be important in motivating them to maintain their weight loss. These provide more in-depth information about their impressions of financial incentives and are detailed under ‘themes regarding financial incentives for maintaining weight’. Themes are presented in relation to the participant group as a whole because no marked differences were evident for gender or weight status (whether a participant had maintained, re-gained or lost further weight), with the exception of strong support for the continued use of meal replacement products by those who had re-gained weight.

#### Maintenance program impressions

There was strong support among focus group participants for a program to assist with weight loss maintenance. Reasons given included the need for ongoing support to remain focussed on keeping up behaviour changes necessary to maintain weight loss. Although the preferred mode of program delivery varied across participants, there were clear individual preferences expressed for receiving information and support from the HWFL team by one of either text message, email, telephone or directly via the program website. Participants also valued being able to track their progress and provided examples of either continuing to use the program online tracking system, using a range of ‘apps’, or wearable physical activity tracking devices.

*Yeah, something to maintain what you’ve achieved through the program … I do think you plateau. You have a really good experience and then it flattens off for a while, and sometimes you probably need that motivation, I don’t know what it would be, to just get off the plateau and go down to the next level, you know.* (Female, 69 years, female-only group).

Conversely, a few participants expressed no desire for ongoing support as they felt they had made sustainable and lifelong changes.

*I think if you prove that you’ve maintained your seventy kilo weight and it hasn’t changed, you’re self-sustainable. I don’t need the group. I’ve changed my behaviour so much that you know what, this is rock solid.* (Male, 65 years, male-only group).

#### Financial incentives impressions

Discussion about financial incentives conveyed a general feeling of distrust and indignation at the prospect of being offered a financial incentive. For many participants, regardless of having maintained their weight loss or not, improved health and wellbeing was regarded as the ultimate incentive for both initial weight loss and maintenance following program completion. For some the concept of a financial ‘reward’ was not specific enough to their health to encourage ongoing behaviour changes. The following excerpt was typical of the sentiment expressed in the groups:

*I think it should be to make you feel better, to reward you that you look better or feel better or exercise better, like whatever the reward is, should be relative to a healthy, better - making yourself feel better, because I think money just doesn’t do it.* (Female, 58 years, female-only group).

Participants expressed varying degrees of support and opposition to the different types of financial incentives as illustrated by quotes in Table 5. While a few participants liked the idea of a cash reward, most did not see value in receiving cash, as it would be absorbed into their general spending. Some discussed the size of the reward and felt that it would need to be large to motivate them to persist with behaviour change. All groups felt that a cash reward would be most meaningful if it was directly linked to reducing their health insurance premium. Some participants were wary of non-cash rewards, particularly retail vouchers which were limiting and might require additional spending to buy what they wanted. The offer of a discount or rebate on HWFL meal-replacement products was more attractive, as they saw this as directly related to achieving weight loss maintenance and therefore improving their health.

**Table 5:** Quotes relating to types of financial incentives

Type of Financial Incentive	Supporting quote	Opposing quote
Cash reward	*... it is a win, win situation, you still keep on losing weight and you earn some cash, so yeah, I guess it could be some encouragement. Because … free money is always a good motivator ... You avoid your own money but you think, oh I’m working very hard for it, this makes you work hard to keep your weight off. Yeah maybe.* (Female, 55 years, female-only group)	*Well, it doesn’t mean that we don’t want to maintain our weight, it just means that that wouldn’t be an incentive, really… What we’re saying is basically, with the services that we would like, it’s like value adding to what we’ve already done. Whereas to me, getting cash is not value adding, right? Value adding is being offered additional things....* (Female, 69 years, female-only group)
Non-cash reward	*There’s merit to it, provided it’s something you wish to ... that is of value to you.* (Male, 67 years, male-only group)	*I find half the time I get vouchers from retail outlets, they’re vouchers I don’t want to use, so I tend to give them away or don’t use them. I have about 5 sitting in my purse at the moment that I left downstairs and found.* (Female, 57 years, mixed gender group)
Deposit contract	There were no quotes to use in support of deposit contracts	*It is, you always think, okay, what happens if I can’t keep my commitment and I don’t lose my weight, so what happens to the money, so it is gone, so I haven’t lost my weight and I lost the money so that would make me even more … Yeah you would go out and buy cake and put on more weight.* (Female, 55 years, female-only group)
Matched deposit contract	*You’re getting some return on your investment, besides losing your weight.* (Male, 71 years, male-only group)	*That’s what it is. It’s a financial contract...You’re going to break it at some stage.* (Male, 72 years, male-only group)
Lottery	*I would be more interested in a bit of fun, but you know …* (Female, 73 years, mixed gender group)	*A lottery just means that you’re not really getting rewarded, only that the winner will.* (Male, 53 years, mixed-gender group)

Deposit contracts were unanimously rejected. Participants expressed a strong dislike for paying a deposit which they may not recoup; and were intensely opposed to a health fund holding their deposit, as they felt that they already “paid enough” in insurance premiums. Matched deposit contracts were slightly better received. Some participants expressed that this approach could be considered an investment in their health, but others viewed it as gambling on their own success and provided insufficient encouragement. Some felt that although this option may not appeal to them personally, it may to younger people. Participants believed that younger people may be more likely to be motivated by money than mid-older people. Others talked about having retired and needing to be careful with their money as they had limited earning capacity. While these participants would have valued a financial incentive that saved them money, they viewed a deposit contract as potentially costing them money and therefore not sufficiently motivating to maintain weight loss. A lottery was perceived as potentially more “fun” or “interesting” than other options, but participants were dubious or cynical of their chances of ‘winning’ and therefore did not consider that it would be enough of an incentive.

Although support for the use of financial incentives for weight loss maintenance was low, a small number of participants expressed interest in some types of financial incentives. However, if financial incentives were to be used, participants favoured combining them with other means of ongoing support rather than in isolation.

#### Themes regarding incentives for maintaining weight

A number of themes were generated through focus group data that arose in response to participants’ impressions of financial incentives. These provided a rationale as to why financial incentives might not be acceptable for them. These fell into four categories: health as an incentive, individual responsibility for health behaviour, program support, and peer support.

- *Health as an incentive*

The primary motive for initially losing weight for many participants was to improve their overall health or functional ability, including to reduce pain from osteoarthritis; both serving as powerful incentives to make the necessary changes to lose weight. This idea was common across all discussions and had equal relevance to weight loss maintenance. Participants also raised improved independence, quality of life and keeping up with activities as important motivators. With improved health a priority for almost all participants, they seemed to have difficulty understanding how financial incentives could work as an effective incentive for them. However, participants recognised individual variation and what might motivate one may not motivate another.

*I think the reward should be to better my own health. I just don’t see getting a reward like that. I mean the reward is a better health, feeling better in yourself that is my reward.* (Female, 73 years, female-only group).

*… what motivated me ... there’s a saying that says "pain is the master we obey". It was a great incentive and I feel better. I like my gardening and I like doing things and you know, I have arthritis and this program helped me ... The weight loss helped and the exercise helped me, so overall, I feel a lot better.* (Male, 65 years, male-only group)

- *Individual responsibility for health behaviour*

Overall, participants seemed to have a sense of pride in, and ownership of, their weight loss and the behavioural changes they had made during the 18-week program. There was a strong feeling that the responsibility for health lay with them as an individual, which seemed to be viewed as synonymous with their ability to ‘stick to the changes’. This was at odds with the idea of financial incentives. They felt that they were, or at least should be, self-motivated rather than relying on external monetary incentives, even if in reality they had not been successful in maintaining their weight loss.

*People should take responsibility for themselves.* (Male, 69 years, male-only group).

*It’s not necessary, because I am self-motivated, but a lot of people aren’t self-motivated, so maybe they do need that.* (Female, 73 years, mixed-gender group)

- *Program support*

Participants valued the range of support provided through the HWFL program, including the provision of information, meal replacement products and online tracking of their weight loss progress. Support from the HWFL team by telephone, email or text message in particular was mentioned as important to their overall positive experience with the program, and their successful weight loss. Specifically, being accountable to the program team and having someone monitoring their progress would reinforce their commitment to make and sustain behaviour changes.

*I definitely need a boost of some kind. Somebody from the main office getting in touch with you. Just to prompt you. To remind you or something.* (Male, 78 years, mixed-gender group).

Some expressed feelings of loss after the HWFL program finished, mostly related to the loss of emotional support and the perceived security provided by the discipline and regular contact received through the program structure and team. Although options to continue tracking their progress and/or initiate contact as necessary were available following the program, most participants did not take this up. It was not clear whether they were unaware of this possibility, or chose not to continue contact.

It seems that the perception of the need for program support was more than practical support for many participants, with emotional support identified as crucial to participants’ ability to maintain behaviour changes made during the program. Without this, a number of participants reverted to old behaviours and found it difficult to maintain their weight loss in the context of expectations and challenges of everyday life such as socialising, family celebrations, changed routines and availability of healthy foods on holidays, work functions and everyday temptations. Yet the desire for emotional support to avoid reverting to old habits was in conflict with the ideal of individual responsibility for sustained health changes as some expressed the need to be accountable to someone else (i.e. support of a maintenance program) in order to avoid regressive behaviour. Hence, dependence on the program co-existed with a general view that people should be responsible for their own health.

*Yeah, I slowly unravelled, you know. Once the program was here, we had that contact …. They gave that support at the time … One step was removed, everything just fell apart*. (Male, 71 years, male-only group).

*I needed to do something … but I found that the follow up, it was finished, it was gone and that was the end of the story. I was a bit disappointed* (Female, 72 years, mixed-gender group*).*

Most participants talked positively about using meal replacement products within the program and many attributed the success of the program to those products. While some participants also expressed the value of other HWFL educational components, disproportionate credit was given to “shakes and soups”. As participants who wish to continue using them following the program need to purchase them, some participants suggested that a potentially worthwhile financial incentives would be to discount these products on an ongoing basis.

*A little bit of follow up would be really good and a discount for the shakes and things would be fantastic* (Female, 72 years, mixed-gender group, put on weight)

- *Peer support*

A number of participants expressed that formalised peer support might be motivating and helpful in maintaining their weight loss. The appeal of peer support seemed to stem from the potential of drawing motivation from others who had shared a common experience. In these discussions, there was acknowledgement of individual preferences, highlighting different opportunities for peer support and that one type of support may suit some but not others. Small-group meetings were suggested as a possible way to facilitate a comfortable environment in which participants could honestly share difficulties they faced with each other as well as ideas for overcoming these barriers.

*Somewhere we could all get to. Then we would motivate each other. I think that would help me. A few people getting together and like you were saying, because I think then you feed off the others motivation, you help each other.* (Female, 73 years, mixed gender group).

*Well, support groups can work. Nothing works for everybody all the time.* (Male, 54 years, male-only group).

Peer mentoring was suggested as an alternate type of support, acknowledging that program participants who had already experienced the challenges of trying of maintain their weight loss might be able to provide valuable advice and support. A few participants felt that an online support network could work for them, offering an easily accessible forum for questions and discussion around managing sustained weight loss. Peer support did not, however appeal to all participants with a few voicing a preference for taking full responsibility for their weight loss maintenance without relying on others.

## Discussion

Our online survey results indicate that the majority (93.9%) of HWFL participants (mid-older Australians with private health insurance) supported a booster or maintenance program, a finding confirmed by our focus group discussions. More than half (56.9%) of survey respondents thought that financial incentives could be useful to support weight loss maintenance and a healthy lifestyle. However, when this idea was explored in more depth during focus groups, the majority of participants doubted the usefulness of financial incentives in the context of maintaining improved healthy behaviours. To our knowledge mid-older adults’ perceptions regarding financial incentives specifically for weight loss maintenance have not been published previously.

Our study supports the findings of previous research that there is low acceptance of financial incentives for healthy lifestyle and disease prevention among older adults [22]. Nonetheless, the divergence between our survey results and the in-depth focus group discussions points to the importance of fully understanding program participants’ preferences about financial incentives. These may differ depending on the complexity and variability of financial incentives in different contexts as outlined by Adams et al. [42], underscoring the importance of canvassing the views of older adults in financial incentive program design using mixed methods [22]. Exploring the acceptability and perceived usefulness of different types of financial incentives as part of formative research is one way of gaining insight to which particular (if any) financial incentives may be worth including in a lifestyle intervention with this population.

Group discussions reflected a general ambivalence towards the use of cash, vouchers or gifts which were not directly related to improving their health. A Canadian study showed high acceptability of voucher-based incentives in older adults [24] and a Singaporean study indicated a preference for cash, followed by supermarket vouchers [43]. While our survey results somewhat reflect these findings, focus group participants expressed concern about the usefulness of vouchers. They felt that unless a cash reward specifically reduced their health insurance premiums it would not be worthwhile. In Australia however, regulations governing competition within the private health insurance sector preclude this option [44].

There was unanimous dislike for, and distrust of, deposit contracts. Focus group participants did not feel that deposit contracts would help with weight loss maintenance, but thought they could possibly appeal to younger people. As participants pay high insurance premiums, they felt that outlaying further money with no guaranteed return was unnecessary. Although little is understood about the acceptability of commitment or deposit contracts in any setting [45], in behavioural economics terms our results may originate from older adults’ preference for decisions with positive emotional outcomes (e.g., enjoyment as opposed to loss), and therefore targeting regret in older adults may not be useful [46]. A lottery was viewed to be more fun than other options but participants perceived that with low chances of winning, it was not sufficiently motivating. These findings add to mixed findings in the literature about the acceptability of health-related lotteries. Although it has been proposed that older adults might find lottery systems attractive [46], our focus group findings are more aligned with the suggestion that cash or shopping vouchers, and not lotteries, might be more acceptable to the general population [25]. Our participants expressed reluctance to ‘gamble’ in relation to both deposit contracts and lotteries; possibly explained by research indicating that age-related cognitive decline is associated with older adults being more risk averse and likely to prefer immediate rewards over delayed but larger rewards [47]. Deposit contracts or lotteries with mid-older adults in this setting, are therefore not likely to be effective intervention tools.

Study participants proposed alternative means of motivating weight loss maintenance and healthy behaviours and these could be broadly grouped as *program support* measures or *formalised peer support* (e.g., support group meetings and peer mentoring opportunities). Participants viewed peer support opportunities as potentially offering emotional and practical support to maintain motivation for sustained behaviour change to achieve weight loss maintenance and overcome barriers, through the sharing of experiences. While *program support* represented services provided by the HWFL program, peer support is not currently included in HWFL service delivery. Our findings align with previous research identifying peer support and education as preferred alternatives to financial incentives [27]. Other peer support research with older adults in lifestyle interventions is limited, with mixed results. For example, an intervention combining financial incentives and peer support, although considered feasible, had no effect on walking outcomes [48]. However, telephone-based peer mentorship, more than financial incentives, was associated with improved glucose control in older African Americans with diabetes [49]. The acceptability and perceived usefulness of peer support shown in our study highlights the merit of further investigation into ways of incorporating peer support into a weight loss maintenance intervention.

The notion of health, and its encompassing benefits, as the most important incentive for sustaining behaviour change and weight loss was common across discussions. Personal responsibility for health was discussed particularly in opposition to the use of financial incentives, where participants felt that motivating improved health was not the obligation of their health insurer and should stem from the individual. Previous qualitative research on the use of financial incentives for health care decisions also found people viewed health as a personal responsibility and that people should be self-motivated to improve their health [28, 50]. While financial incentives may be in conflict with individual responsibility for health [22], Promberger and Marteau [5] found limited evidence that financial incentive rewards actually undermine motivation in physical activity or weight loss interventions. It is possible that although financial incentives are considered a potentially worthwhile health promotion tool, among mid-older adults with private health insurance in our study, improved health had a more intra-personal motivating value.

These findings are similar to those of participants in a Finnish type 2 diabetes prevention intervention where participants were grouped according to how difficult they found the lifestyle change process associated with participating in a weight loss program [51]. This qualitative impact study identified subgroups among participants who fell along a spectrum with positions associated with weight loss or weight gain. Positions ranged from finding lifestyle changes difficult or impossible (primarily expressed by weight-gainers), struggling to balance health and unhealthy choices (mainly weight-gainers but also weight-losers), and finding the process unproblematic and routine (weight-losers). Although our findings fell along a similar continuum, they did not show clear distinctions associated with weight status. In considering personal responsibility and obesity, Brownell and colleagues [52] proposed the value of creating conditions which nurture and sustain personal responsibility, thus allowing individuals to make healthy decisions most easily, as imperative to public health. The challenge to policy makers and program planners in providing support to assist with maintaining weight loss and healthier lifestyles is to balance the tension between the importance placed by some on individual responsibility for health with the need for external support mechanisms expressed by others.

### Strengths and limitations

One strength of this mixed method study is the triangulation of data through quantitative and qualitative methods. It should be noted that Studies A and B did not confirm each other at the broad level of participants’ perceived acceptability and utility of financial incentives for weight loss maintenance. The quantitative responses suggested acceptance and perceived potential utility of financial incentives, whereas the qualitative data reflected more negative views. One possible reason for this is that the nature of surveys allows for only responses bounded by the answer options provided, whereas during a qualitative discussion participants can more thoroughly explore their reaction to financial incentives; alternatively they may feel more inhibited in openly expressing their opinions about accepting financial rewards than in an online survey.

The sample size for Study A was relatively small, but generally representative of the target population profile. Although the socio-economic distribution of participants was similar for Study A (50%) and all health insured Australians (51.5%) [10], Study B was sampled only from Sydney-based participants and therefore most participants (82.1%) were from more socio-economically advantaged areas. The differences in socio-economic distribution between Study A and Study B may have contributed to the variation in views towards financial incentives between the two study groups as financial incentives are known to work better for low-income individuals [53]. While there was a commonality of themes in focus group discussions, perceptions expressed may not cover the range of opinions of all mid-older adults with private health insurance.

The perceptions investigated in this study were those of real-world program participants as opposed to those of the general public as reported by others [26, 27, 30]. As participants self-selected to attend the focus groups, it is possible that their views may differ from those who did not agree to attend. Social desirability bias and peer pressure may have influenced responses of some participants who may have found the group situation intimidating, and might have felt pressure to agree with the dominant view [54, 55]. While some may consider this a limitation, other consider these dynamics as providing external validity to focus group methodology [56]. This group dynamic however, may have been limited for the group which only had two members, but does not necessarily discount their contributions to understanding how participants felt about financial incentives.

## Conclusion

Our study addresses mid-older adults’ perceptions to using financial incentives for the maintenance of weight loss, which have not previously been explored in Australian participants of a health insurance lifestyle program. Although results of an online survey indicated moderate support for the use of health promoting financial incentives, the results of focus group discussions confirm the lack of support among older adults for using health promoting financial incentives [22]; and add to limited existing qualitative research in this context. If financial incentives are to be considered, program planners will need to balance the conflict between participant beliefs about the individual responsibility for health with their desire for external supports to motivate and sustain weight loss maintenance. A possible avenue to achieve this may be to capitalise on the view of participants that improving their health is the principal incentive to maintaining weight loss.

## Additional file

Additional file 1: Survey Questions (Study A) and Discussion Guide (Study B). (PDF 367 kb)

## Abbreviations

ARIA: Accessibility-Remoteness Index of Australia Plus
HWFL team: HWFL Care Support Team
HWFL: Healthy Weight for Life
SEIFA: Socio-Economic Indexes for Areas
