# Supplementary material for: Acceptability of financial incentives for maintenance of weight loss in mid-older adults: a mixed methods study
Source: BMC Public Health. 2018 Feb 13;18:244. doi: 10.1186/s12889-018-5136-z (PMC5811982; doi:10.1186/s12889-018-5136-z)
Supplement: Supplementary file 1 — Survey Questions (Study A) and Discussion Guide (Study B). (PDF 367 kb) [file 12889_2018_5136_MOESM1_ESM.pdf]

Survey questions (Study A)

Investigating options for providing ongoing support

We are keen to ensure you maintain the lifestyle improvements you have made HWFL and that we offer the best health management program we can. Would you be willing to answer some further questions about booster and incentives program?

◉ Yes ○ No

* Would a 6 week "booster" program every 12 to 24 months be a helpful way to keep you on track and focused on your osteoarthritis and weight management?

- ○ Yes - very helpful
- ○ Yes - somewhat helpful
- ○ No opinion
- ○ Not helpful at all

If a "booster" program was available, please rate how important the following elements would be to you.

Access to additional subsidised KicStart VLCD	○ Very important	○ Important	○ Neutral	○ Not important at all
Active follow up, contact and support from the Healthy Weight For Life team	○ Very important	○ Important	○ Neutral	○ Not important at all
Online tools to track your weight and osteoarthritis symptoms	○ Very important	○ Important	○ Neutral	○ Not important at all
Additional written and online resources to help manage personal stress, anxiety or sadness	○ Very important	○ Important	○ Neutral	○ Not important at all
Additional written and online resources to help develop and practice problem solving skills	○ Very important	○ Important	○ Neutral	○ Not important at all

Financial Incentives for Maintaining Weight

Would the provision of a financial incentive (cash or non-cash) 3-6 months after the completion of HWFL be a helpful way to keep you on track with your weight management?

- ○ Yes - Very helpful
- ○ Yes - Somewhat helpful
- ○ No - Not helpful
- ○ No opinion

Financial Incentives for Maintaining Weight

Would the provision of a financial incentive (cash or non-cash) 3-6 months after the completion of HWFL be a helpful way to keep you on track with your weight management?

- ○ Yes - Very helpful
- ● Yes - Somewhat helpful
- ○ No - Not helpful
- ○ No opinion

Some definitions

- A **non-cash reward** is one which is not cash, but has a monetary value – provided to you after a set period of time, if you maintain your weight loss or achieve a weight maintenance goal.
- A **cash reward** is a certain amount of money – provided to you after a set period of time, if you maintain your weight loss or achieve a weight maintenance goal.
- A **deposit contract** involves you making a cash deposit - which is refunded to you at the end of a pre-determined time period, if you maintain your weight loss or achieve a weight maintenance goal.
- A **matched deposit contract** involves you making a cash deposit - which is refunded to you at the end of a pre-determined time period, if you maintain your weight loss or achieve a weight maintenance goal. In addition, the deposit refunded to you is matched so that you "double your money".

Considering the above definitions, please indicate whether you would be motivated by each incentive.

Non-cash reward	○ Strongly agree	○ Agree	○ Neither agree or disagree	○ Disagree	○ Strongly disagree
Cash reward	○ Strongly agree	○ Agree	○ Neither agree or disagree	○ Disagree	○ Strongly disagree
Deposit contract	○ Strongly agree	○ Agree	○ Neither agree or disagree	○ Disagree	○ Strongly disagree
Matched deposit contract	○ Strongly agree	○ Agree	○ Neither agree or disagree	○ Disagree	○ Strongly disagree

What amount of money would motivate you to maintain your weight loss?

- ○ $50
- ○ $100
- ○ $150
- ○ $200
- ○ More than $200
- ○ Other (please specify) ______

If you were to enter into a deposit contract (either matched or unmatched) what do you think should happen to your deposit if you do not meet the weight maintenance goal set?

- ○ Donated to a charity of my choosing
- ○ Donated to any charity
- ○ Other (please specify) ______

What kind of non-cash reward would motivate you to maintain your weight loss?

- ○ Reward points
- ○ Gym membership
- ○ General gift vouchers
- ○ Vouchers for leisure-related products
- ○ Vouchers for food products
- ○ Other (please specify) ______

Do you have any ideas about specific cash or non-cash rewards that would motivate you to maintain the weight you lost during the Healthy Weight for Life program?

Discussion Guide (Study B)

Introductions

Introduce self and explain purpose of project

- The Prevention Research Collaboration at The University of Sydney is undertaking a research project looking at programs and strategies that might be able to assist in maintaining weight loss after the completion of the Healthy Weight for Life program.

Make mention of survey and consent and payment for attending session

Inform the need to audio record the session and mention anyone observing the session; reassure the recording will be used for internal purposes only, and nobody will be individually identified

Explain the importance of everyone contributing to the discussion, particularly if they feel differently to the person next to them and go over the following group rules:

- The most important rule is that only one person speaks at a time. There may be a temptation to jump in when someone is talking but please wait until they have finished.
- There are no right or wrong answers, you can be as frank and open as you like. You do not have to speak in any particular order.
- When you do have something to say, please do so. There are many of you in the group and it is important that I obtain the views of each of you.
- You do not have to agree with the views of other people in the group.
- Does anyone have any questions?
- Please turn off mobiles if possible.

Have participants introduce themselves

Experiences of the Healthy Weight for Life Program

1. We will start by getting your comments on the Healthy Weight for Life Program you have recently completed.
   1.1 How did you feel about the program overall? Was it worth doing?
   1.2 What did you like about the program? What worked for you?
   1.3 Were there parts of the program you didn't like?

Experiences since finishing the Healthy Weight for Life Program

2. How have you been going since the program has finished? (*prompt for weight regain, unhealthy eating, being inactive, etc.*)

3. Have you found it easy or difficult to maintain the lifestyle changes that you made during the Healthy Weight for Life Program?

4. (If difficult) Why is that, what was most difficult? Are there any things you can suggest that could help you maintain those changes?

Maintenance Program

5. I would now like to hear your thoughts about whether you think some kind of maintenance or booster program would be a good idea.

Describe maintenance / booster program SHOW CARD. Ask them to rate their level of interest, acceptability, positives, negatives etc.

6. How do you think such a program could be improved?

Non-Cash Reward Program

7. What are your thoughts about whether a non-cash reward would be a good idea for helping you to maintain your weight loss?

Describe non-cash reward program SHOW CARD. Ask them to rate their level of interest, acceptability, positives, negatives etc.

8. How do you think such a program could be improved?

Cash Reward Program

9. What are your thoughts about whether you think a cash reward would be a good idea for helping you to maintain your weight loss?

Describe cash reward program SHOW CARD. Ask them to rate their level of interest, acceptability, positives, negatives etc.

10. How do you think such a program could be improved?

Deposit Contract Program

11. What are your thoughts about whether a deposit contract would be a good idea for helping you to maintain your weight loss?

Describe deposit program SHOW CARD. Ask them to rate their level of interest, acceptability, positives, negatives etc.

Discuss options for deposits not refunded (e.g. money goes to a charity, money is divided among participants who do achieve weight maintenance or achieve their goal, etc.). Participants might have other ideas.

12. How do you think such a program could be improved?

Matched Deposit Contract Program

13. What are your thoughts about whether a matched deposit would be a good idea for helping you to maintain your weight loss?

Describe matched deposit program SHOW CARD. Ask them to rate their level of interest, acceptability, positives, negatives etc.

14. How do you think such a program could be improved?

Lottery Program

15. What are your thoughts about whether a lottery would be a good idea for helping you to maintain your weight loss?

Describe lottery program SHOW CARD. Ask them to rate their level of interest, acceptability, positives, negatives etc.

16. How do you think such a program could be improved?

Payment Structure Options

17. What type of payment structure do you think you would best motivate you?

Describe payment structure options SHOW CARD. Ask them to discuss the pros and cons of each option.

Combinations – preferred models

18. I would like to ask you to nominate which of the options is your preferred (or closest to your preferred) option. SHOW CARDS

19. In your view, what are the benefits of your preferred option?

20. In your view, what are the risks and/or amendments that might need to be considered if your preferred option is pursued?

21. From what we have spoken about already – should any of the options be considered in conjunction with other options (*e.g. SMS reminders, website use, online social networks, social media*)? Can they be combined in any way?

Explore these questions in detail

Other

If participants ask about how their weight will be measured, discuss the options of objective weight measurement (e.g. GP, practice nurse, at an HCF office) for key times and self-reported weight at other times. Get participants thoughts about options and their suggestions of other options.

Participants might ask what weight loss maintenance means. It might mean keeping off the weight they lost during the HWFL program (either in kg or as a percentage of their starting weight, e.g. not putting on more than 2% of their starting weight). They could also be asked to set a goal about what they would like their target weight to be.

- Any final comments

Many thanks for your time and contribution

Ensure completion of survey – demographic information and ratings for proposed programs

Non-cash Rewards

- A reward that has a monetary value but is not cash
- Provided after a period of time or at different time points
- If weight loss is maintained

Examples:
- Voucher for a retail outlet (sports shop, supermarket, etc.)
- Gym membership

Cash Rewards

- A certain amount of cash
- Provided after a period of time or at a number of time points
- If weight loss is maintained

Deposit Contract

As a *commitment to yourself* to maintain your weight:

you make a monetary deposit to HCF which is refunded to you at certain time intervals or at the end of a period of time.

Matched Deposit Contract

As a *commitment to yourself* to maintain your weight:

- you make a monetary deposit to HCF which is refunded to you at certain time intervals or at the end of a period of time.
- ***PLUS*** the deposit amount is *matched* either at the end of a period of time OR in increments at a number of time points

Lottery

If you maintain your weight loss:

- You go into a draw to win either a cash or non-cash reward
- This might be a single draw or a number of draws over a period of time

Payment Structure Options

- Reward that is paid as a lump sum
- Reward that is paid at certain intervals

Maintenance Program

- A “booster” program
  - To help you maintain the benefits of the Healthy Weight for Life Program (eg. to keep off the weight you lost)
  - To encourage sustainable health outcomes

- Possible components of the program
  - Active follow up from the HWFL team
  - Online tools to track weight and other health outcomes
  - Written and online resources (to help manage stress or to help with problem solving skills)
  - Financial incentives to motivate you to maintain your weight

Phases of Healthy Weight for Life

- **Phase 1**
  - Motivate
  - Week 1-6
- **Phase 2**
  - Consolidate
  - Week 7-12
- **Phase 3**
  - Maintain
  - Week 13-18

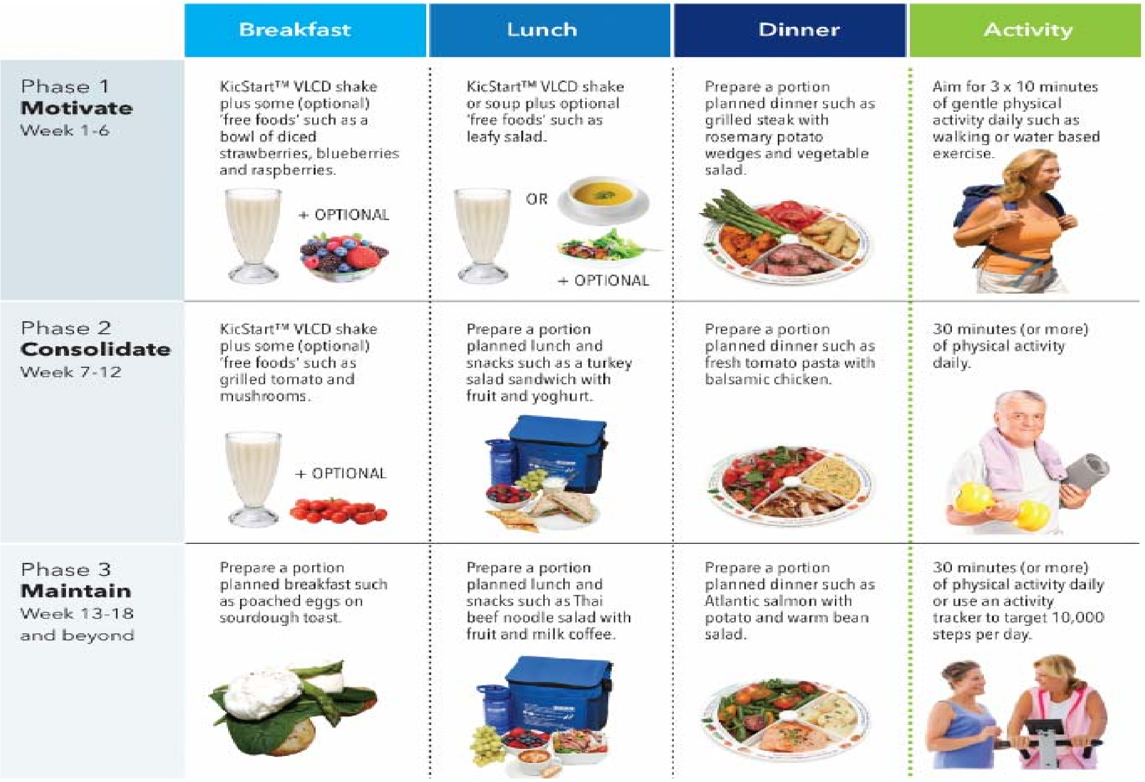

	Breakfast	Lunch	Dinner	Activity
Phase 1 **Motivate** Week 1-6	KicStart™ VLCD shake plus some (optional) 'free foods' such as a bowl of diced strawberries, blueberries and raspberries. + OPTIONAL	KicStart™ VLCD shake or soup plus optional 'free foods' such as leafy salad. OR + OPTIONAL	Prepare a portion planned dinner such as grilled steak with rosemary potato wedges and vegetable salad.	Aim for 3 x 10 minutes of gentle physical activity daily such as walking or water based exercise.
Phase 2 **Consolidate** Week 7-12	KicStart™ VLCD shake plus some (optional) 'free foods' such as grilled tomato and mushrooms. + OPTIONAL	Prepare a portion planned lunch and snacks such as a turkey salad sandwich with fruit and yoghurt.	Prepare a portion planned dinner such as fresh tomato pasta with balsamic chicken.	30 minutes (or more) of physical activity daily.
Phase 3 **Maintain** Week 13-18 and beyond	Prepare a portion planned breakfast such as poached eggs on sourdough toast.	Prepare a portion planned lunch and snacks such as Thai beef noodle salad with fruit and milk coffee.	Prepare a portion planned dinner such as Atlantic salmon with potato and warm bean salad.	30 minutes (or more) of physical activity daily or use an activity tracker to target 10,000 steps per day.
